# Inferior: The Challenges of Gender Parity in the Artificial Intelligence Ecosystem-A Case for Canada

**DOI:** 10.3389/frai.2022.931182

**Published:** 2022-07-01

**Authors:** Lubna Daraz, Bebe S. Chang, Sheila Bouseh

**Affiliations:** ^1^School of Library and Information Science, Faculty of Arts and Sciences, University of Montreal, Montreal, QC, Canada; ^2^University Libraries, Nova Southeastern University, Davie, FL, United States; ^3^Independent Researcher, Hamilton, ON, Canada

**Keywords:** artificial intelligence (AI), women, gender equity, gender parity, labor force, Canada

## Abstract

Artificial Intelligence (AI) systems are gaining momentum in complementing and/or replacing performing tasks typically done with the aid of human ability. AI systems, inherently human creations, are, however, beset by, wittingly or unwittingly, so-called male chauvinism, despite all the advancements made in the progress of civilization to make inroads for women's equitable participation in the labor force, particularly in relation to the digital economy, and more importantly, AI. In regards to the Canadian context, this perspective has examined the evidence to find research highlighting gender representation in the Canadian AI ecosystem. We found a lack of studies on women and their contribution to AI-related activities. Canadian women's participation in their country's AI sector therefore should go beyond mere instruments such as the Montreal Declaration for a Responsible Development of AI, and disjointed interests. On a more general level, the paucity in a paradigm shift toward AI-female friendly policies from design phase to implementation omits the female voice for adequate representation for action. Advocating for Canadian women in the AI sector requires a voice of unison best achieved through parliamentary action. This perspective is thus issuing a clarion call to attaining gender fairness and equity, global principles under the United Nations (UN) Sustainable Development Goals, to which the Government of Canada is committed.

## The Global AI Gender Gap and Canada's Case

Artificial Intelligence (AI) is rapidly becoming the new skill-set of today's Fourth Industrial Revolution, blurring the lines between the physical and virtual workforce. However, achieving gender parity in such a labor market is cause for concern with the World Economic Forum (WEF) finding that females represent just 22% and males, 78% (World Economic Forum, 2018). In its updated Global Gender Gap Index report of 2021, the World Economic Forum downgrades Canada from 14th place in 2006 to 24th place out of 156 countries despite being in first place in educational attainment (World Economic Forum, 2021). Canada is behind developing countries, such as Namibia, Rwanda, Lithuania, Nicaragua, Costa Rica, and the Philippines at 6th, 7th, 8th, 12th, 15th, and 17th places, respectively. A study also examining relative AI skills penetration for the period 2015–2020, finds that men outstripped women in the US and Canada, while India, South Korea, Singapore, and Australia are more successful in closing the gap in achieving equity (Stanford University, 2021).

Canada is an industrialized country advanced in AI technologies and innovations. The 2020 *Pan-Canadian AI Strategy Impact Assessment Report* cites that in 2017, the Government of Canada invested $125 million into developing the world's first national AI approach (CIFAR, 2020). The report states that Canada received an increase of $600 million in funding for AI start-ups, more than doubling 2017's. According to the report, more than 45 companies have invested in Canadian AI research labs since 2017, including Microsoft, Facebook, Thomson Reuters, Google DeepMind and other prominent organizations. Key research and development investments were made by Google Brain's first Canadian lab, Facebook's AI research lab in Montreal, and DeepMind's first international lab in Edmonton. These leads have enhanced Canada's capacity in world-class AI research and training, collaboration, implementation, and attract and retain AI experts and scholars. To ensure the successful implementation of such initiatives, the report notes that the *Montreal Declaration for a Responsible Development of AI* adopted 10 principles including equity, diversity and inclusion to accommodate what is important to individuals and groups (University of Montreal, 2021). The Montreal Declaration has attracted, as of this writing, the signatures of only 2,194 individuals in Canada and around the world and 200 organizations (University of Montreal, 2021). What is surprising is that while the Declaration espouses equity, diversity and inclusion of women, it excludes aboriginal women who symbolize diversity. The Declaration is published in several languages such as Russian, Chinese, English, French, Arabic and German, but not in any of the Canadian Aboriginal languages. The Declaration, therefore, omits any representative interests from Canada's nearly one million aboriginal people of which over 700,000 (over 4% of Canada's population) are women (Statistics Canada, 2016).

Despite these injections of effort, women, particularly underrepresented women of color, still face challenges within the AI sector. For example, it has been reported in 2019 that in Canada only 14% of women are AI researchers (Kiser GaM, 2022). This means women in Canada encounter challenges in participating in AI domains, such as education, research and industry jobs, especially in obtaining leadership positions within organizations. In this perspective, we examine the global gender gap in AI and especially in Canada, followed by a comparison between the US and Canada with further clarification to Canadian women's role in the AI sector.

## Comparison Between USA and Canada

The gap between male and female participation in the AI sector in Canada's immediate southern neighbor, the United States, the world's leading industrialized nation in science and technology, is significantly wide. The World Economic Forum report shows this gap at 72%, a decrease of 2% over 2015 (World Economic Forum, 2018). In the meantime, Canada's gender gap is at 77%, a rise of 3%. Equitable and gender participation disparities in AI skills reflect a similar high rank of 70% (female: 23%; male: 77%) and 69% (female: 24%; male: 69%) for the United States and Canada, respectively. This is despite the countries' rankings as one and five, respectively in AI skills. In terms of the overall gender gap, out of 149 countries, Canada ranks at 24, a drop from 16th place in 2018. Notably, the United States ranks at 30 elevating from its number 51 position in 2018 (World Economic Forum, 2018, 2021). Overall, according to the estimates, it will take 61.5 years to close the gender gap in North America (World Economic Forum, 2021). Women make up 35% of Canadian managers and 42.2% in the United States, while the gap in wages and income is at 30% (World Economic Forum, 2021). In the United States, despite gender parity on the educational front (100%), men outrank women in STEM specializations by three-folds (30%) (World Economic Forum, 2021). The preceding global findings depicting the underrepresentation of female participation in all aspects of AI development and deployment processes permeate all areas, and while they can be repeated across the world, the disparity between genders in leading industrilised and technological counties is, in the least, astonishing.

## Empirical Research on Women's Role in the Canadian AI Sector

Quebec boasts itself as a leader in the world of deep learning and machine learning research. The 2020 *Pan-Canadian Artificial Intelligence Strategy Impact Assessment Report* cites Canada producing over 2,000 AI papers in 2019 alone according to Scopus, an abstraction and citation database under the multibillion-dollar multinational publishing giant, Elsevier (CIFAR, 2020). However, while Canada has produced a plethora of research in AI, a cursory review of the literature in PubMed and Google Scholar has found a paucity of empirical research in gender disparities in AI in Canada. Therefore, we present the challenges for women in AI in Canada from a broader perspective using gray literature found in government documents, white papers, conference abstracts, and reports.

## Canadian Women Under-Representation in the AI Sector

To increase female representation and participation in the AI ecosystem, as well as expand Canada's role as an AI leader in the global economy, it is vital to first identify the potential challenges. Women in Canada like in other developed countries encounter similar barriers such as a lack of equity in opportunities offered by AI innovations.

The 2020 *Pan-Canadian Artificial Intelligence Strategy Impact Assessment Report* does not explicitly emphasize gender considerations in educating and training the next generation of potential AI talents (CIFAR, 2020). Vulnerable populations, including women of different racial and ethnic backgrounds, have limited access to AI education in Canada despite the over $600 million investment in start-ups noted in the Report.

Statistics Canada reported the results of a study that was conducted based on the analysis of the data provided by the Education and Labor Market Longitudinal Platform (ELMLP) (Statistics Canada, 2021). This study included the data about the students who were registered in a science, technology, engineering, and mathematics and computer science (STEM) program in 2010 and analyzed their retention rate during the next few years. According to this report, since women quit the program before finishing it, they are underrepresented in STEM disciplines. The results revealed three vital observations based on retention, persistence and rate of graduation (Wall, 2019). Firstly, as of 2015, fewer women (66%) than men (72%) continued in the STEM programs. Equally, more women (23%) than men (12%) switched majors from a STEM to a BHASE (business, humanities, health, arts, social science, and education) program and fewer women (11%) than men (16%) abandoned undergraduate studies (Wall, 2019). Secondly, more women (82%) preferred to stay longer in engineering compared to men (77%). Similarly, this was compared to 9% of women and men in general and integrated sciences, where many students eventually moved to a more specific STEM program or a BHASE program (Wall, 2019). Finally, the study found that, in all fields of the STEM programs, the rate of graduation was higher for women than men (Wall, 2019). According to the study, within the usual 4 years of a program, more women (27%) than men (16%) graduated with a STEM degree from the computer and information sciences program (Wall, 2019).

Despite women being in the majority in obtaining a university degree (Ferguson, 2016), only 1 in 10 (6.7%) women studied in STEM compared to 3 in 10 of their male counterparts. Since there is a low number of female graduates in AI, it is difficult to find Ph.D. graduates with experience in AI. As a result, women continue to be underrepresented in AI research and innovations in Canada, and they continue to be underrepresented in AI conference publications (Kiser GaM, 2022). Therefore, it is predicted that women in Canada are more likely to leave STEM fields, such as engineering and computer science than men (Catalyst, 2020).

A gender-based study published by Statistics Canada in 2019, comparing occupational pathways for STEM graduates demonstrated that 2 in 10 women were employed compared to 3 in 10 men in a STEM-related occupation with a postsecondary education (Frank, 2019). Moreover, the study reports a growing number of women are leaving STEM occupations after their graduation. Among the potential factors for the exodus are family obligations, marital status, cultural pressure, field of study, a lack of self-confidence, and lack of female role models. These are some of the primary reasons for women not choosing a STEM field for occupation (Clark Blickenstaff, 2005; Dasgupta and Stout, 2014; Frank, 2019).

Furthermore, the lack of graduates in AI may contribute to the gap in women's leadership roles in AI in Canada. However, we see a trend in the increasing number of women in leading influential AI roles spanning different industries in Canada including the academic sector (Johnson, 2018). In addition, it is imperative to note that women leaders may encounter discrimination for not being able to fit in in a male-dominated environment, thus a decline or a lack of interest among women holding a leadership position in AI. It is also essential to highlight the earning disparities for women graduates in Canada. According to Statistics Canada, women graduates earned one-third or 35.8% among all STEM graduates in 2017 in Canada (Catalyst, 2020).

A gap in earning disparities could be another potential reason for women to leave STEM-related occupations. Despite organizations' efforts at recruiting AI talents while improving diversity and inclusion, women continue to face major challenges. For example, women reported consistently encountering challenges during recruitment in AI. They felt that they had to consistently reassert their credibility and expertise when working in a male-dominated work environment (Deloitte, 2022). As a result, there is a lack of motivation in women to retain AI jobs.

Another significant challenge in AI is the female representation in AI development environments. The crux of the AI gender bias challenge lies at the heart of concepts such as who the decision makers and creators of AI technology are (Smith GaR, 2021). *The International Observatory on the Societal Impacts of AI and Digital Technology* (University of Laval, 2022) pointedly characterizes the AI field and digital technology as “a male-dominated environment with very low representation of women and under-represented groups” and notes its attendant adverse “impact on the development of artificial intelligence algorithms that are greatly inspired by their creators.” OBVIA states further that the “conscious and unconscious biases built into these systems can reproduce societal stereotypes and lead to further discrimination.” In fact, the situation commanded a University of Sherbrooke-hosted conference entitled “Social Justice and Artificial Intelligence: Citizen Governance to Reverse Invisibility in Algorithms and Discrimination in their Uses” held in November 2021 (OBVIA, 2021). It should be noted here that OBVIA draws its “ethical charter” from the *Montreal Declaration for a Responsible Development of AI*, which is referred to under Section Comparison Between USA and Canada in this perspective.

The development of AI algorithms should be inclusive and embrace the diversity that by definition includes everyone, especially women and other underrepresented individuals. However, AI technologies are known to be dominated primarily by men, therefore, their influence in the development of AI is unmistakably pronounced. Amazon's “secret AI recruiting tool,” which it had subsequently scrapped, “that showed bias against women” is a testimony to such domination (Dastin, 2018). Premising her explanation of how bias occurs with “We often shorthand our explanation of AI bias by blaming it on biased training data”. Hao (2019) advances three stages at which bias creeps in “long before the data is collected” (Hao, 2019). For example, facial recognition software tends to have gender bias drawbacks. We find difficulties finding research data in Canada to support this claim, however, a study published in *MIT News* reports biases in gender and skin-type in facial analysis systems (Hardesty, 2018). There are groups in Canada led by women who advocate addressing these and other systemic barriers to the inclusion of women of color and all abilities in AI solutions. One such organization is TechGirls Canada, a non-profit hub for Canada's women in the STEM disciplines with Work Finding and Immigrant Women's Prosperity in STEM at the core of its work (TGC, 2022).

## Conclusion

The Artificial Intelligence ecosystem needs to be inclusive. In Canada, there is a significant gender imbalance in AI because of the underrepresentation of women in aspects of education, recruitment, job retention, earnings, leadership roles and AI developments. Like in any other industry, women encounter barriers to integrating and sustaining their participation in AI which has been a predominantly male-dominated industry in Canada.

Different groups of marginalized women, for example, Aboriginals, Black, Muslim, immigrant, Asian, women with different abilities and sexual orientations, are not equitably represented in the AI ecosystem in Canada. Accordingly, many initiatives are needed by governments and women advocacy groups to pave the way for establishing a safe, inclusive and flourishing environment for women. In view of the foregoing points, the role of women in decision-making positions should be made a priority. In general, more Canadian women need to be in decision-making roles by including more of them in senior positions (World Economic Forum, 2021). Occupational gender segregation typically driven by stereotyping the intellectual ability of women with the emphasis on favoring men for the technology industry vs. the computing field could be mended by improving the organizational culture for encouraging more female participation in AI (Bian et al., 2017; Cardador, 2017; World Economic Forum, 2021).

Women empowerment, creating inclusive space, equal opportunities, removing unconscious bias, and a genuine effort to embrace equity, diversity and inclusion in decision-making are keys to truly advancing gender equality in AI in Canada. How these might be achieved lies in the need for crossing over the threshold of male-dominant leaders in order for a changed the mindset for women empowerment in AI to take hold. The evolution of corporate social responsibility since the 1930s should have led to adjustments in hiring practices and roles favoring more women participation in a budding digital economy leaning now more to AI. Now, more recently, and in view of the pervasive nature of technology, there are calls for corporate digital responsibility (CDR) which is “a set of practices and behaviors that help an organization use data and digital technologies in ways that are perceived as socially, economically, and environmentally responsible” (Corporate Digital Responsibility, 2022). Among the CDR Manifesto's seven core principles are “Fair and Equitable Access for All,” “Promote Societal Wellbeing,” and “Accelerate Progress with Impact Economy” all of which have implications for gender parity in the digital economy (Corporate Digital Responsibility, 2022). However, there is the noticeable absence of terms such as ‘gender' and ‘female'. Could this be attributed to the fact that of the manifesto's six co-founders, five are male? The negative perceptions of continuation of male voices in a discussion about promoting a fair, just, equitable and sustainable digital economy need to be reserved urgently to help resolve issues such as those raised by Virginia Eubanks in *Automating Inequality: How High-Tech Tools Profile, Police, and Punish the Poor* and Ivana Bartoletti in *An Artificial Revolution: On Power, Politics and AI*.

## Data Availability Statement

The original contributions presented in the study are included in the article/supplementary material, further inquiries can be directed to the corresponding author.

## Author Contributions

LD conceptualized and co-wrote the manuscript with BC and SB. BC helped revise the revised manuscript. All authors contributed to the article and approved the submitted version.

## Conflict of Interest

The authors declare that the research was conducted in the absence of any commercial or financial relationships that could be construed as a potential conflict of interest.

## Publisher's Note

All claims expressed in this article are solely those of the authors and do not necessarily represent those of their affiliated organizations, or those of the publisher, the editors and the reviewers. Any product that may be evaluated in this article, or claim that may be made by its manufacturer, is not guaranteed or endorsed by the publisher.
